# Complexation of Antimony with Natural Organic Matter: Performance Evaluation during Coagulation-Flocculation Process

**DOI:** 10.3390/ijerph16071092

**Published:** 2019-03-27

**Authors:** Muhammad Ali Inam, Rizwan Khan, Du Ri Park, Sarfaraz Khan, Ahmed Uddin, Ick Tae Yeom

**Affiliations:** 1 Graduate School of Water Resources, Sungkyunkwan University (SKKU) 2066, Suwon 16419, Korea; aliinam@skku.edu (M.A.I.); rizwankhan@skku.edu (R.K.); enfl8709@skku.edu (D.R.P.); 2 Key Laboratory of the Three Gorges Reservoir Region Eco-Environment, State Ministry of Education, Chongqing University, Chongqing 400045, China; Sfk.jadoon@yahoo.com; 3 Key Laboratory of Jiangsu Province for Chemical Pollution Control and Resources Reuse, School of Environmental and Biological Engineering, Nanjing University of Sciences and Technology, Nanjing 210094, China; jamali@njust.edu.cn

**Keywords:** antimony, adsorption, complexation, ferric chloride, natural organic matter, water treatment

## Abstract

The presence of natural organic matter (NOM) in drinking water sources can stabilize toxic antimony (Sb) species, thus enhancing their mobility and causing adverse effects on human health. Therefore, the present study aims to quantitatively explore the complexation of hydrophobic/hydrophilic NOM, i.e., humic acid (HA), salicylic acid (SA), and L-cysteine (L-cys), with Sb in water. In addition, the removal of Sb(III, V) species and total organic carbon (TOC) was evaluated with ferric chloride (FC) as a coagulant. The results showed a stronger binding affinity of hydrophobic HA as compared to hydrophilic NOM. The optimum FC dose required for Sb(V) removal was found to be higher than that for Sb(III), due to the higher complexation ability of hydrophobic NOM with antimonate than antimonite. TOC removal was found to be higher in hydrophobic ligands than hydrophilic ligands. The high concentration of hydrophobic molecules significantly suppresses the Sb adsorption onto Fe precipitates. An isotherm study suggested a stronger adsorption capacity for the hydrophobic ligand than the hydrophilic ligand. The binding of Sb to NOM in the presence of active Fe sites was significantly reduced, likely due to the adsorption of contaminants onto precipitated Fe. The results of flocs characteristics revealed that mechanisms such as oxidation, complexation, charge neutralization, and adsorption may be involved in the removal of Sb species from water. This study may provide new insights into the complexation behavior of Sb in NOM-laden water as well as the optimization of the coagulant dose during the water treatment process.

## 1. Introduction

Antimony (Sb) is a semi-metallic element widely found in the environment that is introduced into water bodies as a result of natural processes and anthropogenic activities [1]. In recent years, higher rates of Sb contamination have been reported in natural water reservoirs near mining and smelting areas [2,3]. For instance, the underground well water near Xikuangshan Sb mine, in China, contained 6000 µg/L Sb [2]. In addition, Sb levels up to 1000 µg/L were detected in the groundwater near abandoned Sb mines in Slovakia [4]. Due to its abundance and toxicity, higher Sb pollution in drinking water supplies is a potential risk to human health [5,6]. Therefore, in order to protect human health, the United States Environmental Protection Agency (USEPA), European Union (EU), and World Health Organization (WHO) have set regulatory standards for Sb of 6, 10, and 5 µg/L, respectively [7]. Furthermore, Korea has also set the standard for Sb in tap and bottled water at 20 and 15 µg/L, respectively [8].

Antimony mobility in the environment is regulated by various advanced technologies including coagulation, membrane separation, ion exchange, adsorption, phytoremediation, and electrochemical processes [9]. As compared to other removal methods, coagulation–flocculation/adsorption processes are the most efficient and cost-effective technique widely used in the drinking water industry [10]. Earlier studies [11,12] have indicated that different sorbents such as activated lignin–chitosan extruded pellets and brown microalgae contain great potential for removing cationic organic pollutants and methylene blue dye from various aqueous media. Previous studies [7,13] have also shown the effectiveness of ferric chloride (FC) coagulant in remediating Sb over aluminum-based coagulant. However, the stability of Sb was greatly influenced in the presence of other dissolved substances such as natural organic matter (NOM) [7,14]. Several types of NOM are ubiquitously present in the aquatic environment, in concentrations ranging from 0.5 to 10 mg C/L [15]. Moreover, they form complexes with metals and may also enhance the mobility and bioavailability of Sb species in natural waters [16,17]. Low molecular weight (LMW) hydrophilic NOM, such as salicylic acid (SA) and L-cysteine (L-cys), is mainly composed of carbon and nitrogenous compounds, such as carboxylic acids, carbohydrates, thiol and other active groups [18,19]. By contrast, high molecular weight (HMW) hydrophobic NOM, such as humic acid (HA) and fulvic acid (FA), mostly contain conjugated double bonds and carboxyl, phenol, hydroxyl, and amine groups with a strong complexation ability with heavy metal ions [20]. Moreover, NOM may participate in redox reactions and form complexes with iron (Fe) surface that increase the transportation of heavy metal ions in water, thus leading to adverse effects on human health [21].

The presence of NOM in Sb-rich water may provide a potential route for human exposure, particularly if the source is being used for drinking water. Previous studies [7,14,22] have shown a significant decrease in Sb removal rate in the presence of NOM during ferric chloride (FC) coagulation. Moreover, total organic carbon (TOC) content has also been reported to be a critical factor in controlling Sb mobilization in water [14]. A recent study [14] indicated that the addition of hydrophobic HA significantly enhanced Sb mobilization in alkaline-contaminated environments. HA molecules have been reported to form stable complexes with Fe surfaces and effectively block Sb from adsorption on hydrous ferric oxide [23]. In addition, it has also been shown that NOM forms Sb–NOM complexes in water, which facilitates the release of Sb in natural and contaminated environments [17]. The formation of Sb–NOM complexes could result from the direct association of Sb with NOM through the replacement of ligands with carboxyl, thiol and phenol groups present in NOM or the inherent metal content of NOM samples [17]. Moreover, it has been reported that at environmentally relevant pH conditions, the organically bound Sb(III) species can be oxidized to Sb(V) species while interacting with humic substances [15]. While some studies [7,14,17,24] have shown the interactions of Sb and hydrophobic NOM in an aqueous environment, the complexation and removal behavior of Sb with NOM is still not well understood. To date, no study has investigated the complexation and removal behavior of Sb with various NOM types, such as hydrophilic and hydrophobic types, during the water treatment process.

Accordingly, the present work aims to quantitatively determine the complexation of Sb with hydrophobic/hydrophilic NOM under different Sb(III, V) concentrations. Moreover, the current study also intends to evaluate the complexation behavior of Sb with NOM following FC coagulation. This study also assesses Sb(III, V) and TOC removal under varying FC doses and NOM concentration by the coagulation–flocculation (C–F) process. Finally, this study also investigates the binding and removal mechanism of Sb in the heterogeneous aquatic environment.

## 2. Materials and Methods

### 2.1. Chemical Reagents and Materials

All chemicals used in the experiments were of reagent grade. The antimony (III) oxide (Sb_2_O_3_), potassium hexahydro-antimonate (KSb(OH)_6_) and NOM (humic acid, salicylic acid, and L-cysteine) were purchased from Sigma-Aldrich (St. Louis, MO, USA). The ferric chloride hexahydrate (FeCl_3_.6H_2_O), nitric acid (HNO_3_), hydrochloric acid (HCl), and sodium hydroxide (NaOH) were obtained from Samchun (Samchun pure Chemicals Co., Ltd., Pyeongteak-si, Korea). The stock and synthetic water solutions were prepared with deionized (DI) water produced using a Milli-Q water purification system (Milli-Q, Millipore Co., Bedford, MA, USA). All glassware and polyethylene bottles were washed with 15% HNO_3_ solution and rinsed with DI water prior to use. In addition, glass vessels were used to store supernatant.

### 2.2. Preparation of Stock Solutions

The stock solutions of 100 mg/L Sb(III) and Sb(V) were freshly prepared by adding Sb_2_O_3_ and KSb(OH)_6_ into 2M HCl and DI water, respectively. The coagulant stock solution of 0.1 M FC was prepared by dissolving FeCl_3_.6H_2_O into DI water. The remaining stock solutions of each NOM, i.e., humic acid (HA), salicylic acid (SA), and L-cysteine (L-cys), were prepared by adding 100 mg powder into 0.1 L DI water. The pH of the HA stock solution was adjusted to 11 by adding 0.1 M NaOH, then stirred at 600 rpm for 24 h so as to improve the solution stability. The suspension was filtered using a 0.45 µm glass fiber filter in order to remove insoluble particles, then the pH was adjusted to 7.0 ± 0.1, and the stock solution was stored in the dark at 5 °C [25]. These model organic substances of different nature (hydrophilic/hydrophobic) were used to simulate different water characteristics. Moreover, these model substances were obtained from commercial sources and have been widely used to provide consistent experimental conditions [17,24,26]. The equivalent TOC concentrations for each NOM, i.e., HA, SA, and L-cys with 10 mg/L were also measured and found to be 4.17, 2.40, and 5.18 mg C/L, respectively.

### 2.3. Complexation Experiments

The interactive behavior of Sb(III) and Sb(V) with each NOM (10 mg/L) in water was studied prior to and after FC coagulation. To this end, complexation experiments were conducted with different Sb(III, V) concentrations (0.1 to 10 mg/L) at neutral pH (7.0 ± 0.1). During the experiments, pH was maintained using 0.1 M HCl or 0.1 M NaOH solutions. The reaction vessels were placed into a 360⁰ rotator (TCLP-601P, Taiwan) for 120 h in darkness with a rotating speed of 27 rpm at room temperature [27].

### 2.4. Laboratory Jar Test Experiments

#### 2.4.1. Jar Test Procedure

The coagulation experiments were conducted using a jar tester (Model: SJ-10, Young Hana Tech Co., Ltd., Gyeongsangbuk-Do, Korea) with six blades at room temperature. Prior to the experiments, a 100 mL solution was added to the beaker and had its pH adjusted to 7.0 ± 0.1 using 0.1 M HCl or 0.1 M NaOH. The coagulation experiments followed three sequential steps as reported in our previous studies [28,29]: (a) rapid mixing at 140 rpm for 3 min; (b) slow mixing at 40 rpm for 20 min, and (c) settling for 30 min. The aliquot was then collected for total organic carbon (TOC) analysis; meanwhile, some volume of the aliquot was filtered using a 0.45 µm glass fiber filter to evaluate the residual concentrations of Fe and Sb species.

#### 2.4.2. Experimental Conditions

For all coagulation experiments, the concentrations of Sb(III, V) species were maintained at 1 mg/L. This set of experiments was performed to investigate the influence of the FC dose (0.1 to 0.3 mM) on Sb(III, V) and TOC removal in the presence of each NOM (10 mg/L). Another set of experiments was also conducted under optimum FC doses to study the effect of each NOM (0 to 20 mg/L) on Sb(III, V) and TOC removal, respectively. The average values were reported for triplicate analysis. 

#### 2.4.3. Isotherm Models

Adsorption data were calculated assuming that the removal of NOM was due to the adsorption onto Fe precipitates. The two most commonly used adsorption isotherm (Langmuir and Freundlich) models were applied to fit the experimental data using non-linear Equations (1) and (2):

(1) $$q_{e}=\frac{q_{m}{\;K}_{L}{\;C}_{e}}{1+{\;K}_{L}{\;C}_{e}},$$

(2) $$q_{e}=K_{F}{\;C}_{e}^{\frac{1}{n}},$$

where q_e_ (mg C/g) and q_m_ (mg C/g) are the equilibrium and maximum adsorption capacity of organic ligands on FC surface sites, respectively; C_e_ (mg C/L) is the equilibrium concentration of organic ligands in solution; K_L_ (L/mg) in the Langmuir equation represents the Langmuir constant related to adsorption energy; and K_F_ (mg C/g) (L/mg)$\frac{1}{n}$ and *n* in the Freundlich equation represent the constants related to the adsorption capacity and intensity of heterogeneity, respectively.

### 2.5. Analytical Methods

The pH of the solution was measured using a pH meter (HACH: HQ40d Portable pH, conductivity, oxidation-reduction potential (ORP) and ion selective electrode (ISE) Multi-Parameter Meter, Hach Company, Loveland, CO, USA). The residual concentration of Fe and total Sb (dissolved Sb + organically bound Sb) were analyzed by inductively coupled plasma optical emission spectrometry (ICP-OES: Model Varian, Agilent technologies, Sana Clara, CA, USA). For the measurement of free Sb species, a high-performance liquid chromatography and then inductively coupled plasma mass spectrometry (HPLC-ICP-MS: Model Agilent 1100, Sana Clara, CA, USA) were used. The NOM bound Sb was determined by calculating the difference between total and free Sb. The detailed procedure can be found elsewhere [30]. The elemental compositions of the studied HA, SA, and L-cysteine were determined using an elemental analyzer (Vario EL III, Elementar, Langenselbold, Hesse, Germany). The TOC content was measured with a TOC analyzer with an ASI-L liquid autosampler (TOC-5000A, Shimadzu Corp, Kyoto, Japan). Finally, Fourier transform infrared spectroscopy (FT/IR-4700, spectroscopy, JASCO Analytical Instruments, Easton, PA, USA) was employed to characterize the functional groups of pristine NOM, Sb-NOM complexes, and FC composite flocs.

## 3. Results and Discussion

### 3.1. Characteristics of NOM

The relative percentage distributions of carbon (C), hydrogen (H), nitrogen (N), oxygen (O), and sulfur (S) of the studied hydrophobic/hydrophilic NOM are presented in Supplementary Material Table S1. The elemental analysis indicated that C and O were the primary elements present in each NOM. The relative C and O contents in HA, SA, and L-cys were 61.29, 35.40, and 27.63%, and 33.95, 20.23, and 24.59%, respectively. In addition, the L-cys also includes the major fraction of S (24.68%) in its molecular structure.

**Figure 1:**
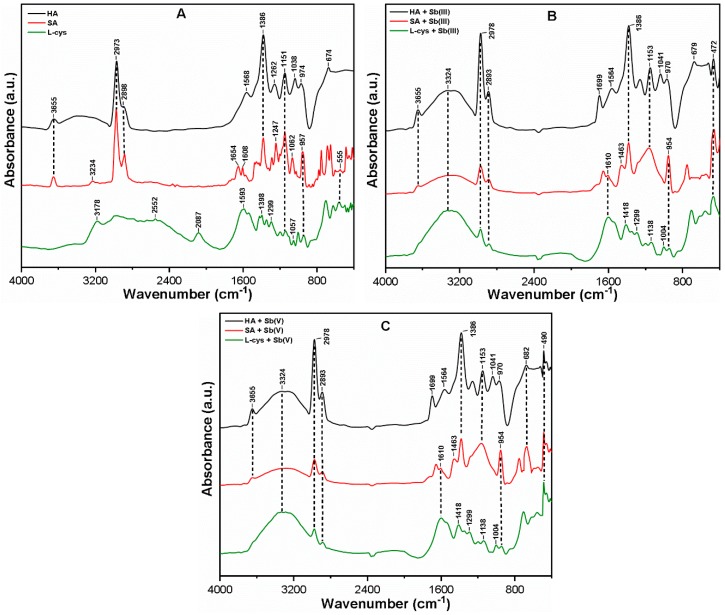
FT-IR analyses of (**A**) pristine HA, SA, and L-cys; (**B**) Sb(III)-NOM; and (**C**) Sb(V)-NOM complexes.

The FT-IR spectra for the studied NOM prior to and after interacting with Sb(III, V) species are presented in Figure 1A–C. The bands that appeared at approximately ~3655 and 3350–3100 cm^−1^ were assigned to the partial N–H and broad OH stretching vibrations [27,28]. The peaks appearing around ~2973 and ~2898 cm^−1^ were ascribed to the asymmetric and symmetric stretching vibrations of the aliphatic C–H and C–H_2_ moiety, respectively [31]. The two small peaks observed at ~2552 and ~2087 cm^−1^ corresponded to the S–H and N–H stretching vibrations [19]. The bands near ~1654 and ~1608 cm^−1^ were attributed to the symmetric and asymmetric stretching vibrations of C=O (COO^−^) [32]. Moreover, the peak in the range 1600–1500 cm^−1^ indicates the presence of ketones with C=O stretching vibrations [27]. The peaks at 1398 (in L-cys), 1386 1299, 1151, and 1262 cm^−1^ were subjected to the symmetric stretching vibrations of COO^−^, CH_3_, S=O, and C–O, and the anti-symmetric stretching vibration of C–O, respectively [33]. The other peaks observed at ~1247, ~1062, ~1038, and ~957 cm^−1^ indicate the presence of C–O stretching along with OH deformation in the COOH and phenolic group, C–O–C stretching in carbohydrate, C–O–C stretching in the ethers Ph–O–C group, and C–O stretching in the carboxylic acid group, respectively [27,34]. Furthermore, the peaks appearing at 674 cm^−1^ and 555 cm^−1^ correspond to C–H bending and C–O–C stretching vibrations, respectively [27].

Figure 1B,C show the FT-IR spectra of Sb(III)-NOM and Sb(V)-NOM complexes, respectively. Following the interaction of HA with both Sb(III, V) species, the small peak that appeared at 1699 cm^−1^ generally indicates the formation of the Sb-HA complex through C=O stretching of COOH and other carbonyl groups [35]. Moreover, the shift in the peak from ~674 to ~679 cm^−1^ indicates the oxidation of Sb(III) to Sb(V) caused by the bending vibrations of N–H, COOH, and CH groups [36]. The interaction of both Sb species with SA resulted in the disappearance of multiple peaks in the region of 1350–1000 cm^−1^, and a broad band appeared at ~1153 cm^−1^ as well, which indicated the interaction of the Sb species with a C–O group to form a metal ion complex [37]. The disappearance of the peak at ~2552 cm^−1^ in the case of L-cys indicates the formation of covalent bonds between thiols and Sb(III, V) species [38]. In addition, the band at ~1057 disappeared, suggesting the involvement of the C–O–C stretching bond in its reactions with both Sb species. The spectrum of each NOM after interacting with Sb(III, V) species also illustrated a broad wide peak at ~3324 cm^−1^, which is ascribed to the vibration of phenolic groups in the Sb-NOM complex [39]. The presence of a peak around ~1400 cm^−1^ in each NOM further suggests the formation of a Sb-NOM complex through C=C stretching in the aromatic ring and OH bending in phenols [37]. The peaks at ~472 cm^−1^ in Figure 1B and ~490 cm^−1^ in Figure 1C indicate the attachments of Sb(III) and Sb(V) species, respectively [29], after the interaction of organic matter with each Sb species. It is worth noting that the interaction of hydrophilic ligands with Sb(III) species did not oxidize the Sb(III) species to Sb(V). This may be attributable to the presence of LMW organic compounds with reduced oxygen content (Supplementary Material Table S1) and a highly reactive S–H group [40]. However, the presence of oxygen-containing functional groups such as carboxylic acids, carbonyl, phenols, alcohols, ethers, and esters in hydrophobic ligands may provide a pathway for Sb(III) binding and oxidation via electron pair donation to the Sb(III) molecules [17,41]. The oxygen-containing functional groups in NOM may thus play a significant role in the transformation of Sb species in the aquatic environment. 

### 3.2. Sb Binding to NOM

**Figure 2:**
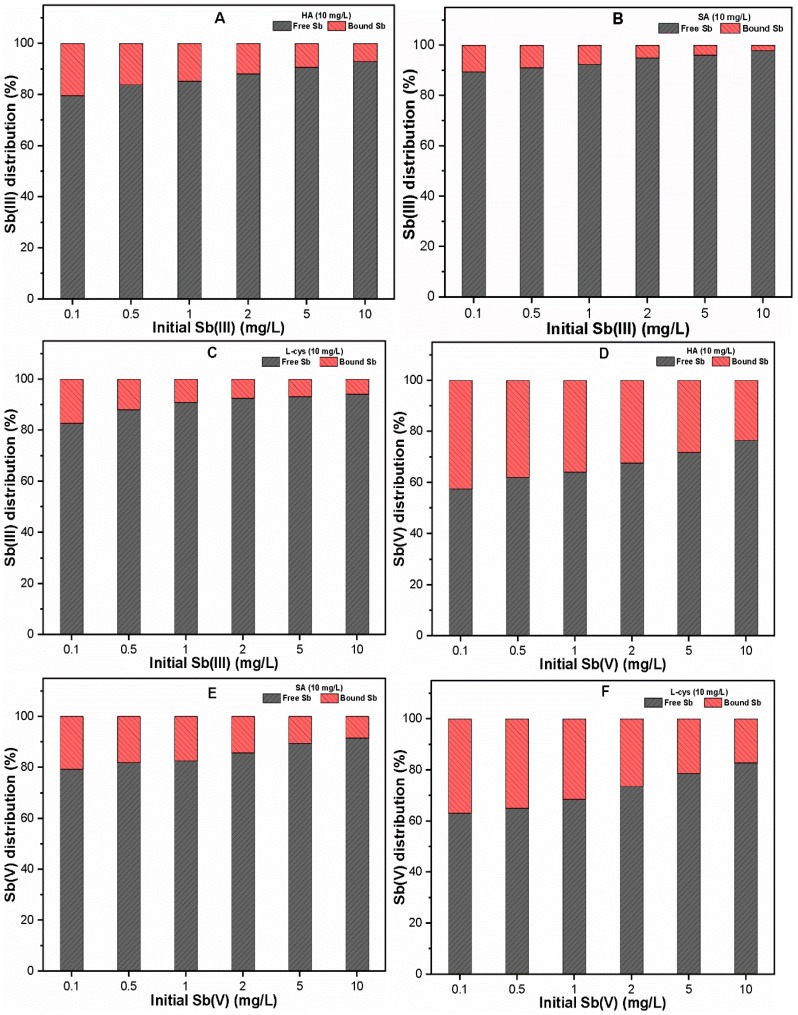
Percent distributions of free and bound (**A**–**C**) Sb(III); and (**D**–**F**) Sb(V) in the presence of 10 mg/L NOM.

In order to further explore the complexation of Sb with each NOM, free and bound Sb were analyzed, and the results are presented in Figure 2A–F. It can be seen that a considerable portion of Sb was complexed with different NOM with higher Sb(V) binding affinity as compared to Sb(III). As shown in Figure 2A,D, the portions of Sb(III) and Sb(V) bound to HA were 7.11–20.4% and 23.48–42.51%, respectively. Meanwhile, the complexation abilities of Sb(III) and Sb(V) species with SA, L-cys were found to be 2.11–10.63%, 5.86–17.31% and 8.43–20.62%, 17.24–36.96%, respectively (Figure 2B,C,E,F). Moreover, Figure 2A–F also illustrate that the proportion of Sb bound with each NOM decreased with increasing initial Sb(III, V) concentration.

The complexation of Sb with NOM has been associated with various functional groups in HA, SA, and L-cys through mechanisms such as ligand exchange and the formation of a negatively charged complex. Moreover, the stabilization of bound Sb occurs via chelation, H-bridges, or cationic metals [17]. The chemical composition of NOM may also affect the Sb complexation ability because trace amounts of metals, including Fe, Si, Mg, and Mn, act as bridging metals during complexation [42]. In addition, the complexation behavior may also depend upon the speciation of Sb(III, V) species. For the pH of interest (7 ± 0.1), the Sb(III) and Sb(V) species exist as Sb(OH)_3_ and Sb(OH)_6_¯, respectively [29]. The presence of carboxylic, phenolic, and amine groups in HA and SA may be responsible for complexation with Sb(III) via a ligand exchange reaction at the Sb center, and thereby, the release of OH^−^ radicals in solution [17]. Moreover, the presence of thiol functional groups in L-cys may result in the formation of the Sb(III)-L-cys complex [43,44,45]. However, strong binding was observed between Sb(V) species and NOM (Figure 2D–F), which may be attributed to the higher positive charge at the Sb(V) center, chelation, and stabilizing effect [46]. In addition, the complexations of negatively charged Sb(V) and negatively charged NOM may also occur via cationic binding with NOM, with a greater extent at a higher metal content [42,47]. Another possible explanation for this could be characteristics of humic substance, as the obtained humic acid was 99% pure containing trace amount of metals, i.e., Fe, Si, Ba, Cr, Mg and Mn, thus these species may interact with Sb and may form heterogeneous complexes such as humic acid-cation-antimony [25]. Our findings are consistent with those of previous studies [27,48,49], which reported stronger As(V) binding with NOM as compared to As(III) species.

Based on the experimental results, it may be suggested that the hydrophobic/hydrophilic NOM may form a complex with Sb(III) and Sb(V) with different binding affinities. Such complexation of Sb species with NOM could facilitate Sb mobility to drinking water sources, and may thus increase the associated health risks as well. Therefore, it is essential to investigate the effect of hydrophobic and hydrophilic NOM on Sb removal at environmental relevant conditions by FC coagulation.

### 3.3. Influence of NOM on Sb and TOC Removal by FC Coagulation

#### 3.3.1. At Various FC Doses

**Figure 3:**
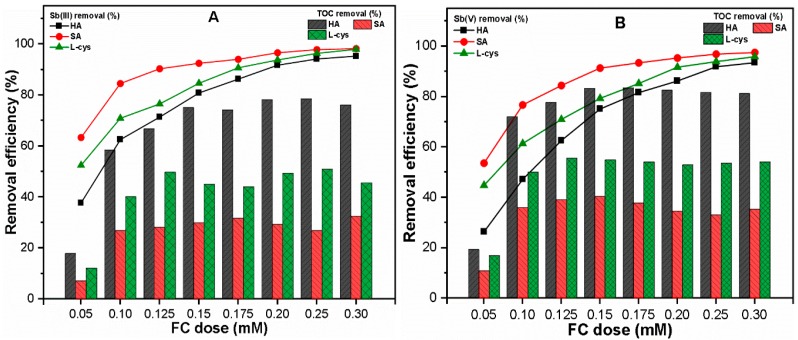
Removal efficiencies of (**A**) Sb(III); (**B**) Sb(V) and respective TOC from solution as a function of coagulant dose.

Figure 3A,B illustrate the effect of NOM on Sb(III, V) and TOC removal under various FC doses, i.e., 0.05–0.30 mM. The results indicated that the FC dose required for Sb(III) removal was relatively less than that for Sb(V) removal. It was considered that the Fe precipitation may play an important role in such discrepant removal behavior, so the Fe precipitates were also measured during the coagulation process. The results revealed that the presence of NOM has an insignificant effect on Fe precipitation in both Sb(III, V) systems (Supplementary Material: Figure S1A,B). Our results are consistent with those of previous studies [7,14,24], which reported higher Sb(III) removal than Sb(V) at the same coagulant dose under similar conditions. The optimum doses (OD) for Sb(III) and Sb(V) under the influence of SA, L-cys were found to be 0.125, 0.175 mM and 0.15, 0.20 mM, respectively. Moreover, HA presented a higher OD for Sb(III) and Sb(V) at 0.20 and 0.25 mM, respectively (Figure 3A,B). Under OD, the removal efficiencies of Sb(III) and Sb(V) in the presence of HA (91.68% and 91.82%, respectively), SA (90.29% and 91.30%, respectively) and L-cys (90.68% and 91.66%, respectively) were observed. The higher FC dose required in a hydrophobic solution may be related to the higher aromatic content in those waters [18,40]. Moreover, the presence of thiol groups in L-cys could be involved in complexation with Sb species [17,43,45], thus requiring a higher FC dose as compared to SA in order to achieve a similar removal efficiency. These results suggest that the Sb removal highly depends on the coagulant dose, type of Sb species, and characteristics of NOM present in water during the C–F process.

The TOC removal was also monitored for both the Sb(III) and Sb(V) systems, as shown in Figure 3. The results suggest that a relatively higher TOC removal efficiency was observed in the Sb(V) system than the Sb(III) system. This may be attributable to the fact that Sb(V) species bound to NOM more strongly than Sb(III) species (Figure 2A–F). Moreover, at OD, the HA showed much higher TOC removal (84.91 and 88.66%) in the Sb(III) and Sb(V) systems, respectively. The higher anionic binding sites in HA might form insoluble complexes with cationic Fe precipitates at an appropriate FC dose as a result of charge neutralization [26,50]. Such phenomena may be responsible for the efficient TOC removal from water, irrespective of Sb species. As compared with hydrophobic ligands, the hydrophilic NOM such as L-cys, SA presented less TOC removal, specifically, 47.80, 30.60% and 57.55, 43.83% in the Sb(III) and Sb(V) systems, respectively (Figure 3A,B). The low TOC removal in SA may be related to the presence of weaker acidic groups and LMW compounds, which hinder the interaction with Fe precipitates [51]. Besides its hydrophilic characteristics, the complexations of positive charge Fe precipitates with a negatively charged S–H group in L-cys might have contributed to its relatively better TOC removal than SA [18]. Our results are consistent with those of previous studies [52,53], which showed higher removal of hydrophobic NOM than hydrophilic during the coagulation process. Our findings revealed that the NOM type is likely to influence the required coagulant dose, and thereby the Sb(III, V) and TOC removal from water during the C–F process. 

#### 3.3.2. At Various NOM Concentrations

**Figure 4:**
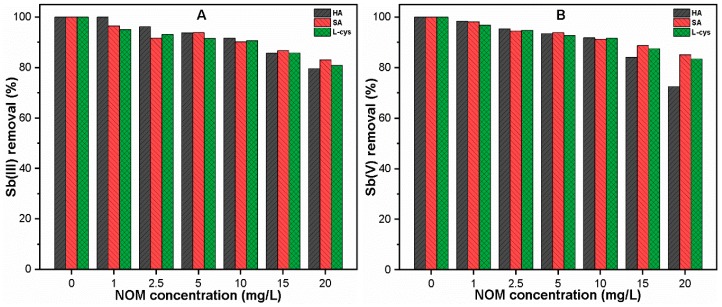
Under optimum FC doses, (**A**) Sb(III); and (**B**) Sb(V) removal at various NOM concentrations (0–20 mg/L) is shown.

Figure 4 shows the effect of hydrophobic/hydrophilic NOM concentration (0–20 mg/L) on the Sb(III, V) removal with optimum FC doses. The complete removal of both Sb(III, V) species was observed in the absence of NOM. However, the presence of a low concentration (0–5 mg/L) of NOM was shown to slightly decrease the removal efficiencies of both species, which continue to decrease upon further increases in concentration. The removal efficiency of Sb(V) was significantly affected by NOM as compared to that of Sb(III). At concentrations ranging between 1–10 mg/L, the hydrophobic ligands presented better Sb(III, V) removal than hydrophilic ligands (Figure 4). By contrast, higher concentrations (15 and 20 mg/L) of hydrophobic ligands remarkably reduced the removal efficiencies of both Sb species. This may be attributable to the dissolved ligand molecules with strong complexation ability with metal ions, thus enhancing the Sb solubility [17,18]. Moreover, the active sites of precipitated Fe responsible for Sb adsorption may be occupied by various functional groups present in HA, resulting in significantly reduced Sb removal at a higher NOM concentration [23]. The Fe precipitation was also monitored at various NOM concentrations, and the results showed nearly complete Fe precipitation (Supplementary file: Figure S2A,B), hence confirming the insignificant effect of NOM on Fe precipitation. 

**Figure 5:**
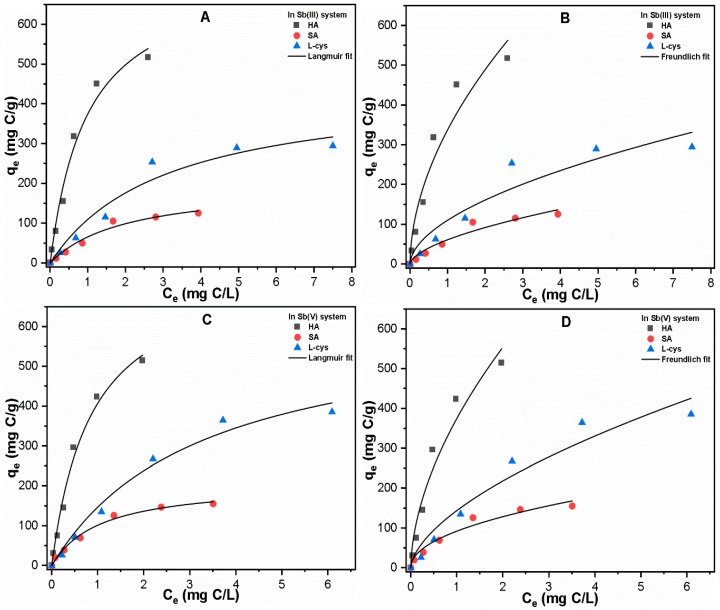
Langmuir and Freundlich fitting of NOM adsorption data in (**A**,**B**) Sb(III); and (**C**,**D**) Sb(V) systems at pH 7 ± 0.1.

In order to further confirm the adsorption phenomena of each NOM onto precipitated Fe, an adsorption isotherm study was conducted at varying NOM concentrations (0–20 mg/L) in the Sb(III, V) system (Figure 5A–D). As shown in Table 1, the maximum adsorption capacity of HA onto Fe precipitates was found to be much higher, at 742.49 and 759.73 mg C/L in Sb(III) and Sb(V) systems, respectively, as compared to those of SA (198.74 and 208.76 mg C/L) and L-cys (446.75 and 627.51 mg C/L). These results suggest the higher adsorption potential of hydrophobic ligands toward Fe precipitates as compared to hydrophilic ligands. Our results are consistent with those of a previous study [54] showing the stronger adsorption capacity of HA on Fe_3_O_4_ surface. In addition, the correlation coefficient (R²) indicated better Langmuir fitting of all NOM on Fe precipitates in both systems, thus suggesting that there is monolayer adsorption onto Fe precipitates. In general, these results suggest that the characteristics of NOM should be considered for the treatment of heavy metal-polluted water.

### 3.4. Sb Binding to NOM during FC Coagulation

**Figure 6:**
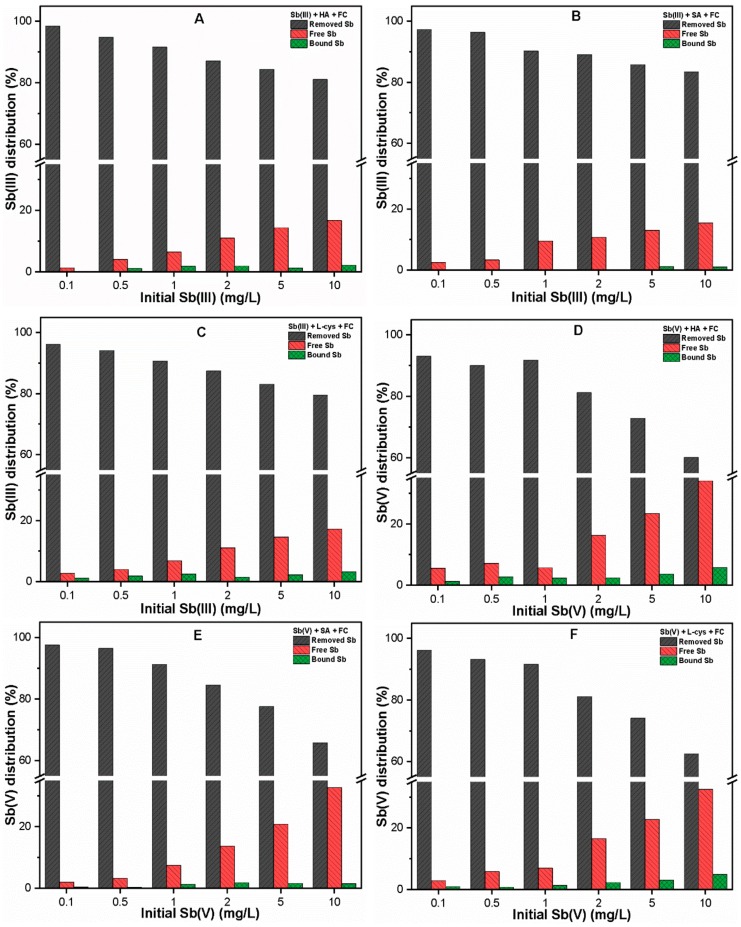
Percentage distributions of (**A**–**C**) Sb(III) and (**D**–**F**) Sb(V) in NOM (10 mg/L) solutions after FC coagulation.

Figure 6 illustrates the percentage distribution of complexed Sb with NOM following FC coagulation. The results indicate that the extent of Sb binding to each NOM was significantly reduced as compared to those of the complexation experiments without FC coagulant (Figure 2). Since the OD was selected for each Sb system, the high removal of both Sb species could be related to the availability of sufficient Fe precipitates (Supplementary Material: Figure S3A,B), thus leaving less free Sb species in solution. Another possible reason is that the adsorption of NOM and Sb-NOM complexes onto precipitated Fe surface sites may result in reduced Sb binding with NOM [16,25]. However, the extent of binding also depends upon the characteristic of NOM, type of Sb species, and metal content present in water. For instance, the hydrophobic ligands with higher aromatic content and hydrophilic ligands with surface reactive thiol groups presented relatively higher Sb binding as compared to the SA molecules with the weaker acidic group. Consistent with our previous observation (Figure 2), Sb binding was more pronounced in Sb(V) species (Figure 6D–F) than Sb(III) species (Figure 6A–C). Moreover, the removal efficiencies of both Sb(III, V) species in the absence and presence of various NOM at optimum FC dosage are also presented in Table S2. Previous studies [55,56] also showed that the sorptive surface (in our case, Fe precipitates) could break the bond of the organic complex (organically bound Sb), leading to enhanced NOM concentration in an aqueous environment. 

It is worth noting that the complexation abilities of Sb(III, V) with NOM (HA, SA, and L-cys) could play a significant role in determining the underlying mechanism involved in the interactions among the ternary system (Sb, NOM, and Fe precipitates). Moreover, the competitive inhibition of Sb adsorption onto Fe precipitates could be expected in the presence of NOM with stronger inhibition in the case of HA, thus leading to the release of more Sb free ions in the aqueous environment (Figure 6A,D). Therefore, in order to protect human health from toxic Sb effects, Sb binding to NOM during the C–F process should be considered.

### 3.5. Mechanism Involved in Sb Removal

**Figure 7:**
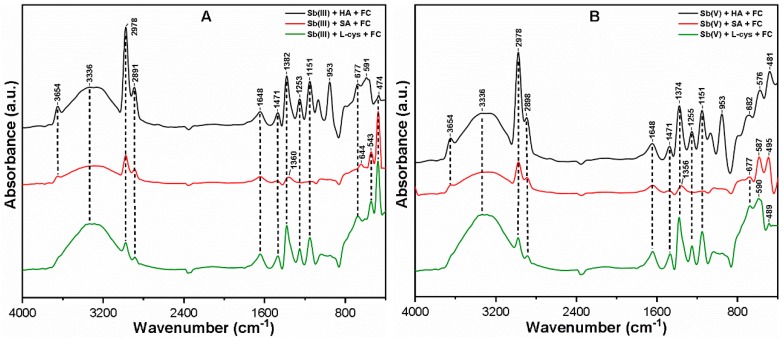
FT-IR spectra of FC composite flocs in the ternary system containing (**A**) Sb(III); (**B**) Sb(V) species at OD after the C–F process.

Figure 7 shows the FT-IR spectra of FC composite flocs after interacting with Sb(III, V) and NOM (HA, SA, and L-cys). The broad peak at ~3336 cm^−1^ present in all flocs corresponds to the stretching vibrations of Fe hydrolyzed products, which form complexes with NOM molecules and Sb species [19,57]. The disappearance of the S–H group at ~2548 cm^−1^ in both Sb(III, V) systems was subjected to covalent bonds between thiols and Sb species onto Fe surface [38]. The peaks appearing at ~1648, and ~1471 cm^−1^ were attributed to the C=O stretching of amide groups and CH_2_ stretching vibrations, respectively [32]. This confirms the adsorbed Sb-NOM complexes onto Fe precipitates with intensities in the order HA > L-cys > SA. The shift in the band to ~1253 cm^−1^ in the presence of HA and L-cys suggests the involvement of carboxylic, phenolic, and thiol groups in complexation with Sb(III, V) via ligand exchange [17]. As shown in Figure 7A, the oxidation of Sb(III) to Sb(V) was confirmed in the presence of HA and L-cys as indicated by the peak at ~677 cm^−1^, which is near ~682 cm^−1^ and assigned to Sb(V)–O stretching vibrations [29]. It should also be noted that the HA molecules contain strong oxidation potential for Sb(III) species, as confirmed from the peak appearing at ~591 cm^−1^ (corresponding to Sb(V) species), while a small peak was observed at ~474 cm^−1^ that was ascribed to Sb(III)-O stretching vibrations [29]. On the other hand, two broad peaks at ~474 and ~543 cm^−1^ were observed in the cases of SA and L-cys, respectively, which correspond to Sb(III)-O stretching vibrations. Consistent with our observation, a previous study [17] also found evidence of Sb(III) oxidation in the presence of HA molecules. 

Thus, the significant shift and disappearance of distinct peaks in the FC composite flocs confirm the complex formation of various contaminants in the ternary system. The oxidation phenomena may also be responsible for the interaction of Sb species with NOM, followed by charge neutralization and adsorption onto active Fe surface sites. The characteristics of flocs supported the removal mechanism of Sb species in the ternary environment well, and can be proposed to be the combination of oxidation, complexation, charge neutralization, and adsorption. However, the removal mechanism may vary with the nature and type of NOM and Sb species present in water.

## 4. Conclusions

In this study, we investigated the complexation behavior of Sb(III, V) species with different NOM, i.e., hydrophobic (HA) and hydrophilic (SA and L-cys) in water. Furthermore, the removal, as well as the binding affinity of Sb, was also quantitatively evaluated in the presence of each NOM following FC coagulation. Our results demonstrated that hydrophobic ligands could form stronger complexes with Sb than hydrophilic ligands, with higher affinity for Sb(V) than Sb(III) species. The optimum FC dose was found to be higher for the removal of Sb species in the presence of HA. In addition, the TOC removal was also found to be higher than hydrophilic ligands, thus indicating increased competition of HA molecules with Sb for Fe binding sites. The adsorption of NOM onto the Fe surface indicated better fitting with the Langmuir model with the maximum adsorption potential for HA molecules. Moreover, the complexation capability of Sb with NOM was remarkably suppressed due to the adsorption of contaminants on Fe precipitates. Furthermore, the removal mechanism involved in Sb removal during the C–F process may be the combination of oxidation, complexation, charge neutralization, and adsorption, as indicated by the FT-IR analysis of FC composite flocs. These results suggest that the characteristics of NOM may influence the fate, mobility, and coagulation performance of Sb species in the drinking water treatment process. 

## Figures and Tables

**Table 1:** Adsorption isotherm fitting parameters of HA, SA, and L-cys on Fe precipitates in Sb(III) and Sb(V) systems.

NOM Type	Sb Charge	Langmuir Fitting			Freundlich Fitting		
		*K_L_* (L/mg)	*q_m_* (mg C/g)	R^²^	*K_F_* $\;(\mathrm{mg}\;C/g)\;(L/\mathrm{mg})\;\frac{1}{n}$	*n*	R^²^
HA	Sb(III)	1.023	742.49	0.978	338.29	1.87	0.932
	Sb(V)	1.159	759.73	0.986	375.42	1.78	0.952
SA	Sb(III)	0.496	198.74	0.971	61.50	1.71	0.939
	Sb(V)	0.948	208.76	0.988	92.44	2.09	0.962
L-cys	Sb(III)	0.326	446.75	0.952	110.61	1.83	0.903
	Sb(V)	0.306	627.51	0.980	144.20	1.66	0.945

## Supplementary Materials

The following are available online at https://www.mdpi.com/1660-4601/16/7/1092/s1, Figure S1: Fe precipitation in (A) Sb(III); and (B) Sb(V) system as a function of coagulant dose; Figure S2: Under optimum FC doses, showing Fe precipitation in (A) Sb(III); and (B) Sb(V) system at various NOM concentrations (0-20 mg/L); Figure S3: At various Sb concentrations (0.1–10 mg/L) and NOM (10 mg/L) showing Fe precipitation in (A) Sb(III); and (B) Sb(V) system after FC coagulation; Table S1: Elemental composition and relative percentage distribution of various NOM; Table S2. Removal of Sb species with and without NOM under various Sb(III, V) concentrations (0.1–10 mg/L) at optimum FC doses.
