# Supplementary material for: Complexation of Antimony with Natural Organic Matter: Performance Evaluation during Coagulation-Flocculation Process
Source: Int J Environ Res Public Health. 2019 Mar 27;16(7):1092. doi: 10.3390/ijerph16071092 (PMC6480550; doi:10.3390/ijerph16071092)
Supplement: Supplementary file 1 [file ijerph-16-01092-s001.pdf]

International Journal of
Environmental Research and Public Health

Supplementary File: Complexation of Antimony with Natural Organic Matter: Performance Evaluation during Coagulation-Flocculation Process

Muhammad Ali Inam[1], Rizwan Khan[1], Du Ri Park[1], Sarfaraz Khan[2], Ahmed Uddin[3], and Ick Tae Yeom[1,*]

[1] Graduate School of Water Resources, Sungkyunkwan University (SKKU) 2066, Suwon 16419, Korea; aliinam@skku.edu (M.A.I.); rizwankhan@skku.edu (R.K.); enfl8709@skku.edu (D.R.P.)

[2] Key Laboratory of the Three Gorges Reservoir Region Eco-Environment, State Ministry of Education, Chongqing University, Chongqing 400045, China; Sfk.jadoon@yahoo.com (S.K.)

[3] Key Laboratory of Jiangsu Province for Chemical Pollution Control and Resources Reuse, School of Environmental and Biological Engineering, Nanjing University of Sciences and Technology, Nanjing 210094, China; jamali@njust.edu.cn (A.U.)

* Correspondence: yeom@skku.edu; Tel.: +82-31-299-6699

Received: date; Accepted: date; Published: date

3. Results and Discussions

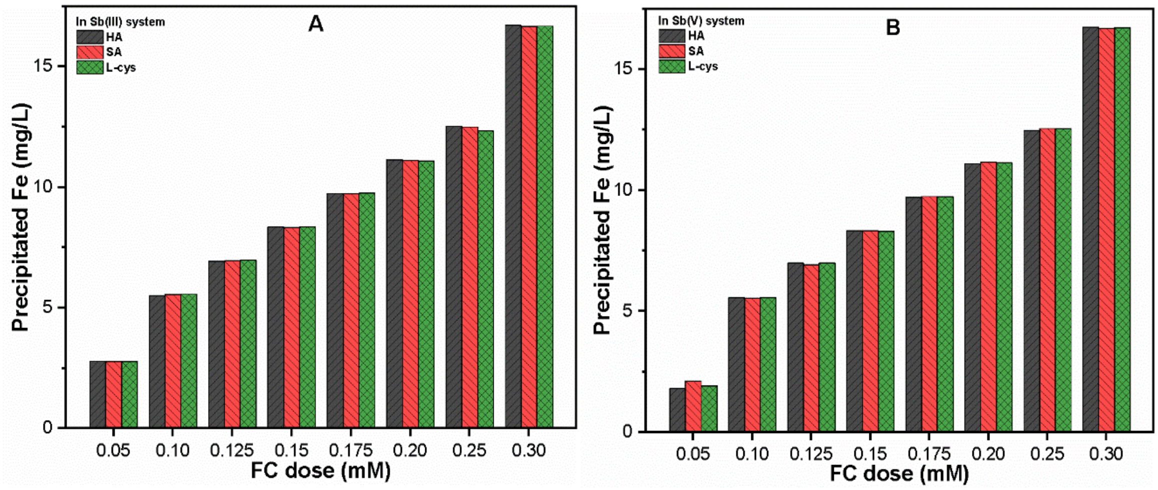

Figure S1. Fe precipitation in **(A)** Sb(III); and **(B)** Sb(V) system as a function of coagulant dose.

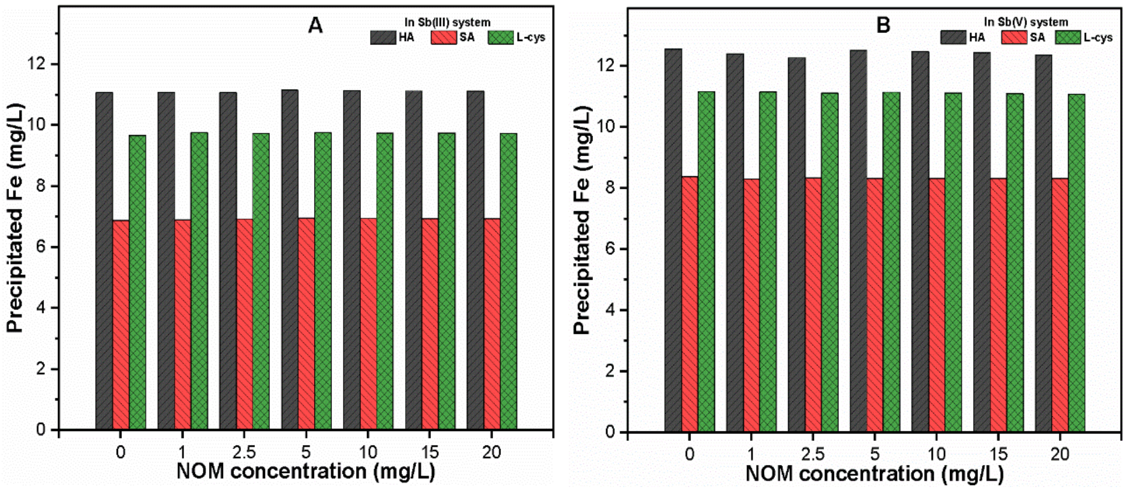

Figure S2. Under optimum FC doses, showing Fe precipitation in **(A)** Sb(III); and **(B)** Sb(V) system at various NOM concentration (0-20 mg/L).

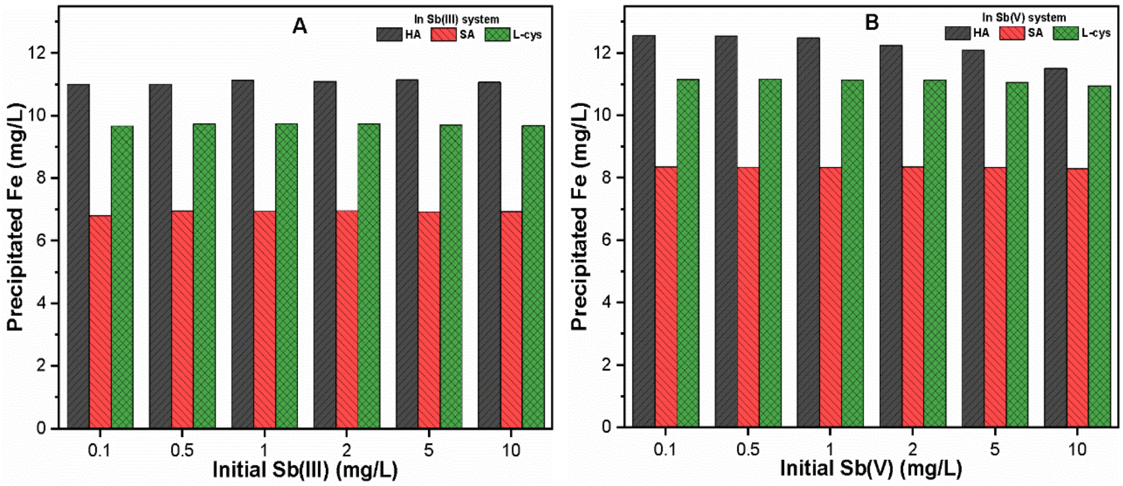

Figure S3. At various Sb concentration (0.1-10 mg/L) and NOM (10 mg/L) showing Fe precipitation in **(A)** Sb(III); and **(B)** Sb(V) system after FC coagulation.

Table S1. Elemental composition and relative percentage distribution of various NOM.

NOM type	Elemental Analysis (%)				
	C	H	N	O	S
Humic acid	61.29	4.52	1.32	31.95	0.63
Salicylic acid	35.40	3.65	0.98	20.23	0.49
L-cysteine	27.63	5.45	7.59	24.56	24.68

Table S2. Removal of Sb species with and without NOM under various Sb(III, V) concentration (0.1-10 mg/L) at optimum FC doses.

NOM		Sb species		Removal (%)
Type	Concentration (mg/L)	Type	Concentration (mg/L)	
Without NOM	0	Sb(III)	0.1	84.81
			1	90.40
			5	78.37
			10	72.37
		Sb(V)	0.1	90.46
			1	89.06
			5	60.30
			10	30.19
Humic acid	10	Sb(III)	0.1	98.54
			1	91.68
			5	84.42
			10	81.15
		Sb(V)	0.1	93.10
			1	91.82
			5	72.90
			10	60.15
Salicylic acid	10	Sb(III)	0.1	97.36
			1	90.29
			5	85.84
			10	83.48
		Sb(V)	0.1	97.54
			1	91.30
			5	77.58
			10	65.74
L-cysteine	10	Sb(III)	0.1	96.15
			1	90.67
			5	83.15
			10	79.54
		Sb(V)	0.1	96.18
			1	91.65
			5	74.15
			10	62.54

© 2019 by the authors. Submitted for possible open access publication under the terms and conditions of the Creative Commons Attribution (CC BY) license (http://creativecommons.org/licenses/by/4.0/).
